# Electrocardiographic Left Ventricular Hypertrophy and Outcome in Hemodialysis Patients

**DOI:** 10.1371/journal.pone.0035534

**Published:** 2012-04-17

**Authors:** Seung Jun Kim, Hyung Jung Oh, Dong Eun Yoo, Dong Ho Shin, Mi Jung Lee, Hyoung Rae Kim, Jung Tak Park, Seung Hyeok Han, Tae-Hyun Yoo, Kyu Hun Choi, Shin-Wook Kang

**Affiliations:** 1 Department of Internal Medicine, College of Medicine, Yonsei University, Seoul, Korea; 2 Severance Biomedical Science Institute, Brain Korea 21, Yonsei University, Seoul, Korea; University of Perugia, Italy

## Abstract

**Background and Aims:**

Electrocardiography (ECG) is the most widely used initial screening test for the assessment of left ventricular hypertrophy (LVH), an independent predictor of cardiovascular mortality in patients with end-stage renal disease (ESRD). However, traditional ECG criteria based only on voltage to detect LVH have limited clinical utility for the detection of LVH because of their poor sensitivity.

**Methods:**

This prospective observational study was undertaken to compare the prognostic significance of commonly used ECG criteria for LVH, namely Sokolow-Lyon voltage (SV) or voltage-duration product (SP) and Cornell voltage (CV) or voltage-duration product (CP) criteria, and to investigate the association between echocardiographic LV mass index (LVMI) and ECG-LVH criteria in ESRD patients, who consecutively started maintenance hemodialysis (HD) between January 2006 and December 2008.

**Results:**

A total of 317 patients, who underwent both ECG and echocardiography, were included. Compared to SV and CV criteria, SP and CP criteria, respectively, correlated more closely with LVMI. In addition, CP criteria provided the highest positive predictive value for echocardiographic LVH. The 5-year cardiovascular survival rates were significantly lower in patients with ECG-LVH by each criterion. In multivariate analyses, echocardiographic LVH [adjusted hazard ratio (HR): 11.71; 95% confidence interval (CI): 1.57–87.18; P = 0.016] and ECG-LVH by SP (HR: 3.43; 95% CI: 1.32–8.92; P = 0.011) and CP (HR: 3.07; 95% CI: 1.16–8.11; P = 0.024) criteria, but not SV and CV criteria, were significantly associated with cardiovascular mortality.

**Conclusions:**

The product of QRS voltage and duration is helpful in identifying the presence of LVH and predicting cardiovascular mortality in incident HD patients.

## Introduction

Cardiovascular disease is prevalent and the most common cause of morbidity and mortality in patients with end-stage renal disease (ESRD) [1]. Even though coronary artery disease and arrhythmia are not uncommon, left ventricular hypertrophy (LVH) is the most frequent cardiovascular manifestation in these patients [2], [3]. LVH is known to be present in more than 70% of incident ESRD patients and increases the risk for cardiac ischemia and congestive heart failure in patients on dialysis [4], [5]. In addition, LVH is a very strong independent predictor of cardiovascular mortality not only among patients with hypertension but also among ESRD patients [6]–[9].

LVH in ESRD patients is mainly attributed to hypertension and anemia [10], [11]. However, accumulating evidence shows that volume overload, arteriovenous fistula, hyperparathyroidism, and oxidative stress also play a role in the pathogenesis of LVH in dialysis patients [12]–[15]. Moreover, LVH regression by modifying these risk factors is associated with improved all-cause and cardiovascular survival [7], while progression of LVH has independent prognostic value for cardiovascular events in dialysis patients [8]. Therefore, early identification of LVH and aggressive treatment to regress LVH should become an important part of management for ESRD patients.

To date, several imaging modalities, such as echocardiography, magnetic resonance imaging (MRI), and computerized tomography, have been performed to detect LVH [16]–[18]. In general, however, electrocardiography (ECG) is more widely used for the assessment of LVH [19]. ECG is a noninvasive, convenient, inexpensive, and easily reproducible test, but the clinical utility of traditional, purely voltage-based ECG criteria for the detection of LVH is limited due to poor sensitivity [20]. Therefore, criteria based on the combination of voltage and QRS duration have been developed and have improved the sensitivity for LVH in the hypertensive population [21], [22]. In addition, several studies elucidated the relationship between LVH based on different electrocardiographic criteria and echocardiographic LVH [23]. Moreover, a very recent study demonstrated the impact of LVH determined by different ECG criteria on clinical outcome in chronic kidney disease [24]. However, little is known about the association between electrocardiographic and echocardiographic LVH in patients with CKD and ESRD. Furthermore, no study has explored whether the prognostic power of ECG varies in ESRD patients based on the diagnostic criteria for LVH. In this prospective study, therefore, we compared commonly used ECG criteria for LVH to ascertain their prognostic significance and investigated the association between echocardiographic LV mass and ECG-LVH criteria in incident hemodialysis (HD) patients.

## Methods

### Ethics statement

The study was carried out in accordance with the Declaration of Helsinki and approved by the Institutional Review Board of Yonsei University Health System Clinical Trial Center. We obtained informed written consent from all participants involved in our study.

### Patients

For this prospective observational study, we initially recruited a total of 603 patients who consecutively started maintenance HD at Yonsei University Health System, Seoul, Korea, between January 2006 and December 2008 and were regularly followed-up at the outpatient clinic. Echocardiography was not performed in 35 patients due to noncompliance or other personal reasons, and in 44 patients it was undergone before the initiation of HD. Of these patients, 207 patients were also excluded for the following reasons: age <18 years (n = 4) or >75 years (n = 11), previous history of peritoneal dialysis or kidney transplantation before HD (n = 139), severe systolic dysfunction (ejection fraction <30%, n = 7), severe valvular heart disease (n = 11), underlying malignancy (n = 21), decompensated liver cirrhosis (n = 3), complete bundle branch block (n = 9), and pacemaker insertion (n = 2). Thus, a total of 317 patients were included in the final analysis.

### Data collection

Demographic and clinical data at the time of HD initiation, including age, gender, and comorbidities, were recorded. The results of the following laboratory tests performed at the same time were also collected: hemoglobin, serum albumin, total cholesterol, calcium, phosphate, and high-sensitivity C-reactive protein levels. The single-pool Kt/V in HD patients was measured with standard Gotch equations on the mid-dialysis day near at the time of discharge [25].

### ECG

Upon admission, a standard 12-lead electrocardiogram was recorded using a MAC 5500 machine (GE Medical system, Milwaukee, WI, USA). All of the patients underwent an additional ECG on a nondialysis day, within 24 hours after the last HD and near the time of discharge, and this follow-up ECG was used for analysis. The output from the MAC 5500 provided QRS duration, PR interval, QT interval, and axes in an automated fashion, but not QRS voltage of every lead. Thus, two independent technicians measured the voltage and discrepancies of >2 mm were resolved by a third reader. The cut-off point for LVH by Sokolow-Lyon voltage (RV5/6+SV1) was ≥35 mm and by Cornell voltage (RaVL+SV3) was ≥28 mm in men and ≥20 mm in women [21]. Products of QRS duration multiplied by the Cornell voltage combination (with 6 mm added in women) ≥2440 mm•msec and by the Sokolow-Lyon voltage combination ≥3674 mm•msec in men and ≥3224 mm•msec in women were used to determine LVH [22]. The QTc interval was calculated based on Bazett's formula: QTc interval = QT/√RR, and the following criteria were used to determine QTc interval prolongation: QTc≥460 msec in women; QTc≥450 msec in men [26].

### Echocardiography

Echocardiography was performed at the time of follow-up ECG based on the imaging protocol recommended by the American Society of Echocardiography using a SONOS 7500 (Philips Ultrasound, Bothell, WA, USA). LV systolic function was defined by LV ejection fraction (LVEF) using a modified biplane Simpson's method from the apical two- and four-chamber views. LV mass (LVM) was determined using the method described by Devereux and Reichek [27], and LV mass index (LVMI) was calculated by dividing LVM by body surface area (BSA). Echocardiographic LVH was defined as a LVMI>131 g/m^2^ for men and >100 g/m^2^ for women [28]. Hypertrophy was considered concentric if LV relative wall thickness was >0.43, and patients with normal LV mass were considered to have normal LV geometry if relative wall thickness was ≤0.43 or to have concentric remodeling if relative wall thickness was increased [23]. Left atrial volume was assessed by the biplane area-length method from the apical two- and four-chamber views and was indexed for BSA. Mitral inflow was assessed with Doppler echocardiography from the apical four-chamber view, and pulse-wave tissue Doppler imaging of the septal mitral annulus was also obtained from the apical four-chamber view. Systolic RV pressure was calculated using the modified Bernoulli equation [4×(tricuspid systolic jet)^2^+10 mmHg].

### Outcome measures

All the patients included in this study were followed-up every 3 months at the outpatient clinic, and all deaths and hospitalizations were recorded in the serious adverse events database. For this study, the mortality events were retrieved from the database and carefully reviewed. Cardiovascular mortality, the primary study endpoint, was considered to be death from myocardial infarction or ischemia, congestive heart failure, pulmonary edema, and cerebral hemorrhage or vascular disorder.

### Statistical analysis

Statistical analysis was performed using SPSS version 18.0 (SPSS Inc, Chicago, IL, USA). Continuous variables were expressed as mean ± SD, and categorical variables as percentages. To determine differences between the two groups, Student's t-test or Mann-Whitney U test was used for continuous variables and the chi-square test was used for categorical variables. Pearson's correlation analysis was performed to estimate the association between LVMI and ECG criteria measurements. Cumulative survival curves were generated by the Kaplan-Meier method, and between-group survival was compared by a log-rank test. The independent prognostic values of electrocardiographic and echocardiographic LVH for cardiovascular mortality were ascertained by Cox proportional hazards regression analysis, which included only the significant variables in univariate analysis. Factors of specific interest were also included in another multivariate analysis. However, each ECG-LVH or echocardiographic criterion was entered separately because there was a significant interaction with each other. The hazard ratios (HRs) and 95% confidence intervals (CIs) were calculated using the estimated regression coefficients and standard errors. The positive predictive values for echocardiographic LVH and cardiovascular mortality were also analyzed by receiver operating characteristic (ROC) curve analysis with calculated area under the ROC curve (AUC). The correlation coefficients between LVMI and each ECG criterion measurements and the AUC of each ECG-LVH criterion for echocardiographic LVH and cardiovascular mortality were compared using a two-tailed Z-score. P-values less than 0.05 were considered statistically significant.

## Results

### Clinical and biochemical characteristics

The baseline patient characteristics according to the presence ECG-LVH are shown in Table 1. The mean age was 56.7±14.2 years (range: 18–75 years), and 53.6% were male. Of the 317 patients, LVH was present in 60 patients (18.9%) by Sokolow-Lyon voltage (SV), 43 (13.6%) by Sokolow-Lyon voltage-duration product (SP), 38 (12.0%) by Cornell voltage (CV), and 43 (13.6%) by Cornell voltage-duration product (CP) criteria. The proportion of male patients was significantly higher in the ECG-LVH group by SV criteria, whereas the proportion of patients with hypertension was significantly higher in the ECG-LVH group by SP criteria (P<0.05). In addition, there was a significant difference in body mass index (BMI) between patients with and without ECG-LVH only by SV criteria (22.5±3.1 vs. 23.8±3.4 kg/m^2^, P = 0.008). Moreover, patients with ECG-LVH by CP criteria showed a significantly higher prevalence of coronary artery disease (P<0.05). A history of coronary revascularization was significantly more prevalent in patients with ECG-LVH by each criterion than in those without ECG-LVH (P<0.05). Blood urea nitrogen, creatinine, albumin, and cholesterol concentrations and Kt/V were also comparable between patients with and without ECG-LVH by each criterion.

**Table 1:** Clinical and biochemical characteristics according to the presence or absence of electrocardiographic LVH.

	Sokolow-Lyon voltage LVH			Sokolow-Lyon product LVH			Cornell voltage LVH			Cornell product LVH		
	No (n = 257)	Yes (n = 60)	P	No (n = 274)	Yes (n = 43)	P	No (n = 279)	Yes (n = 38)	P	No (n = 274)	Yes (n = 43)	P
Age (years)	56.4±14.3	58.3±13.8	0.36	56.0±14.2	61.5±13.0	0.018	56.6±14.4	58.0±12.1	0.57	56.5±14.4	58.4±12.6	0.42
Male gender	130 (51%)	40 (67%)	0.024	144 (53%)	26 (61%)	0.33	152 (54%)	22 (58%)	0.40	144 (53%)	26 (61%)	0.33
Diabetes	143 (56%)	31 (52%)	0.58	149 (54%)	25 (58%)	0.65	151 (54%)	23 (61%)	0.46	148 (54%)	26 (61%)	0.43
Hypertension	227 (88%)	54 (90%)	0.71	239 (87%)	42 (98%)	0.045	245 (88%)	36 (95%)	0.21	240 (88%)	41 (95%)	0.14
Coronary artery disease	39 (15%)	15 (25%)	0.07	44 (16%)	10 (23%)	0.24	46 (17%)	8 (21%)	0.48	41 (15%)	13 (30%)	0.01
History of smoking	36 (14%)	12 (20%)	0.24	40 (15%)	8 (19%)	0.50	43 (15%)	5 (13%)	0.72	40 (15%)	8 (19%)	0.50
Primary renal disease			0.54			0.77			0.88			0.52
Diabetes	143 (56%)	30 (50%)		149 (54%)	24 (56%)		150 (54%)	23 (61%)		147 (54%)	26 (61%)	
Glomerulonephritis	24 (9%)	4 (7%)		26 (10%)	2 (5%)		25 (9%)	3 (8%)		24 (9%)	4 (9%)	
ADPKD	7 (3%)	1 (2%)		7 (3%)	1 (2%)		7 (3%)	1 (3%)		6 (2%)	2 (5%)	
Others	83 (32%)	25 (42%)		92 (34%)	16 (37%)		97 (35%)	11 (29%)		97 (35%)	11 (26%)	
Body mass index (kg/m^2^)	23.8±3.4	22.5±3.1	0.008	23.7±3.4	22.8±3.2	0.11	23.7±3.5	22.8±2.5	0.12	23.6±3.4	22.9±3.4	0.20
MBP (mmHg)	99.0±14.2	100.5±14.4	0.48	99.1±14.3	100.8±13.9	0.47	98.8±14.0	103.2±15.3	0.08	99.3±14.1	99.4±14.9	0.97
Heart rate (bpm)	73.5±13.5	75.0±13.5	0.44	74.1±13.6	72.4±12.8	0.46	73.7±13.7	75.0±12.6	0.58	74.1±13.4	72.3±14.1	0.42
Blood urea nitrogen (mg/dl)	93.7±32.1	93.4±28.8	0.95	93.3±31.2	95.7±33.3	0.64	94.3±32.2	88.9±25.8	0.32	93.1±31.4	97.2±32.3	0.42
Creatinine (mg/dl)	9.8±4.7	9.9±4.2	0.86	9.8±4.6	9.7±4.3	0.92	9.9±4.7	9.1±3.5	0.32	9.7±4.7	10.2±4.1	0.55
eGFR (ml/min/1.73 m^2^)	6.5±3.0	6.3±2.4	0.71	6.5±3.0	6.3±2.5	0.73	6.5±2.9	6.4±2.7	0.82	6.5±3.0	6.1±2.6	0.37
Calcium (mg/dl)	7.8±1.1	8.0±1.1	0.18	7.8±1.1	8.0±1.1	0.18	7.8±1.1	8.1±0.9	0.07	7.8±1.1	7.9±1.2	0.56
Phosphate (mg/dl)	6.2±1.9	6.3±1.7	0.85	6.2±1.9	6.3±1.8	0.78	6.2±1.9	6.1±1.9	0.80	6.2±1.8	6.5±2.1	0.32
Albumin (g/dl)	3.5±0.7	3.6±0.6	0.33	3.5±0.7	3.6±0.5	0.33	3.5±0.7	3.4±0.6	0.26	3.5±0.7	3.5±0.6	0.95
Cholesterol (mg/dl)	161.9±50.0	155.3±41.2	0.34	161.3±49.3	157.0±43.1	0.60	159.0±48.2	173.0±48.8	0.10	160.1±49.0	164.2±45.1	0.61
Hemoglobin (g/dl)	8.4±1.6	8.6±1.3	0.56	8.4±1.6	8.7±1.4	0.34	8.4±1.6	8.8±1.5	0.16	8.4±1.6	8.6±1.6	0.55
C-reactive protein (mg/l)	0.8±0.9	1.1±1.5	0.18	0.8±1.1	0.8±1.0	0.91	0.8±1.1	0.8±0.9	0.88	0.8±1.1	1.0±1.2	0.40
Single-pool Kt/V	1.3±0.3	1.2±0.3	0.76	1.1±0.4	1.2±0.3	0.79	1.2±0.4	1.3±0.3	0.71	1.2±0.3	1.3±0.3	0.81
No of antihypertensive drugs	2.1±1.2	2.2±1.2	0.55	2.1±1.2	2.1±1.1	0.89	2.1±1.2	2.4±1.1	0.13	2.1±1.2	2.2±1.0	0.79
Antihypertensive drugs												
β-blockers	144 (56%)	30 (50%)	0.40	151 (55%)	23 (54%)	0.84	152 (55%)	22 (58%)	0.69	152 (56%)	22 (51%)	0.60
ACE inhibitors or ARBs	148 (58%)	39 (65%)	0.29	160 (58%)	27 (63%)	0.59	158 (57%)	29 (76%)	0.02	159 (58%)	28 (65%)	0.38
Calcium channel blockers	191 (74%)	47 (78%)	0.52	205 (75%)	33 (77%)	0.79	206 (74%)	32 (84%)	0.17	203 (74%)	35 (81%)	0.30
Statin use	74 (29%)	17 (28%)	0.94	74 (27%)	17 (40%)	0.09	77 (28%)	14 (37%)	0.24	72 (26%)	19 (44%)	0.02
Coronary revasculization	31 (12%)	16 (27%)	0.004	31 (11%)	16 (37%)	<0.001	37 (13%)	10 (26%)	0.03	33 (12%)	14 (33%)	<0.001
Stroke	16 (6%)	7 (12%)	0.14	20 (7%)	3 (7%)	0.94	19 (7%)	4 (11%)	0.41	19 (7%)	4 (9%)	0.58

ADPKD, autosomal dominant polycystic kidney disease; MBP, mean arterial blood pressure; BPM, beat per minute; eGFR, estimated glomerular filtration rate; No, number; ACE, angiotensin converting enzyme; ARB, angiotensin II receptor blocker; LVH, left ventricular hypertrophy.

Data are presented as n (%) or mean ± SD.

### Echocardiographic and electrocardiographic findings

As shown in Table 2, LV mass index (LVMI) and left atrial volume index were significantly higher, while LV ejection fraction was significantly lower in patients with LVH by each criterion compared to those without ECG-LVH. In addition, the early mitral inflow velocity to peak mitral annulus velocity (E/E') ratio was significantly higher only in patients with ECG-LVH by QRS voltage-duration product criteria. On the other hand, QT interval was significantly prolonged in patients with ECG-LVH based on SP and CP criteria than in those without ECG-LVH by these criteria.

**Table 2:** Echocardiographic and electrocardiographic parameters according to the presence or absence of echocardiographic LVH.

	Sokolow-Lyon voltage LVH			Sokolow-Lyon product LVH			Cornell voltage LVH			Cornell product LVH		
	No (n = 257)	Yes (n = 60)	P	No (n = 274)	Yes (n = 43)	P	No (n = 279)	Yes (n = 38)	P	No (n = 274)	Yes (n = 43)	P
**Echocardiography**												
Ejection fraction (%)	62.2±10.1	57.8±10.5	0.003	62.0±10.0	57.0±11.6	0.003	61.8±10.0	58.4±12.1	0.05	62.3±9.7	55.7±12.1	0.001
LVEDD (mm)	51.5±5.2	53.5±5.5	0.011	51.5±5.0	54.4±6.3	0.001	51.8±5.0	52.9±7.2	0.34	51.5±5.0	54.3±6.7	0.01
IVS thickness (mm)	10.7±1.8	11.6±1.7	<0.001	10.8±1.8	11.7±1.6	0.001	10.8±1.7	11.9±2.2	<0.001	10.7±1.7	12.0±2.1	<0.001
PW thickness (mm)	10.6±1.6	11.5±1.6	0.001	10.7±1.7	11.5±1.5	0.004	10.7±1.6	11.7±1.6	0.001	10.6±1.6	11.8±1.4	<0.001
LV mass index (g/m^2^)	127.3±27.7	145.0±26.3	<0.001	127.7±27.2	149.7±27.9	<0.001	128.1±27.5	149.5±26.9	<0.001	127.2±27.0	152.6±26.7	<0.001
LA volume index (ml/m^2^)	34.6± 12.2	40.8±12.7	<0.001	34.6±12.0	43.6±13.0	<0.001	35.2±12.4	39.8±12.5	0.032	34.9±12.2	41.2±13.0	0.002
RV pressure (mmHg)	31.7±10.6	33.4±11.2	0.28	31.7±10.4	34.0±12.2	0.22	31.8±10.8	33.8±10.1	0.31	31.6±10.4	34.5±12.1	0.12
E/E'ratio	16.6±7.1	18.1±8.0	0.17	16.5±6.9	19.6±9.1	0.046	16.3±6.9	21.1±8.1	<0.001	16.4±6.7	19.8±9.6	0.033
Echocardiographic LVH	169 (54%)	49 (60%)	0.017	181 (53%)	37 (65%)	0.009	184 (53%)	34 (69%)	0.003	181 (53%)	37 (70%)	0.009
Relative wall thickness	0.42±0.08	0.43±0.07	0.18	0.42±0.08	0.43±0.07	0.59	0.42±0.07	0.45±0.10	0.12	0.42±0.08	0.44±0.08	0.05
Concentric remodeling/LVH	105 (41%)	31 (52%)	0.13	113 (41%)	23 (53%)	0.13	113 (41%)	23 (61%)	0.019	112 (41%)	24 (56%)	0.07
**Electrocardiography**												
PR interval (msec)	168.5±30.6	163.8±25.4	0.28	167.4±30.1	169.2±27.3	0.73	168.7±30.1	160.3±25.6	0.11	167.0±28.9	171.9±34.3	0.33
QRS duration (msec)	93.0±14.7	94.0±11.2	0.63	91.4±11.9	104.7±20.4	<0.001	93.0±14.2	94.6±13.2	0.50	91.3±13.4	105.1±12.6	<0.001
QTc interval (msec)	458.0±35.6	467.0±40.8	0.12	457.3±35.7	475.1±39.7	0.003	458.8±35.2	466.6±46.1	0.22	457.1±33.3	476.5±50.9	0.019
ST-T abnormalities	17 (7%)	13 (22%)	<0.001	16 (6%)	14 (33%)	<0.001	15 (5%)	15 (40%)	<0.001	12 (4%)	18 (42%)	<0.001
Sokolow-Lyon voltage (mm)	23.8±6.3	41.3±5.4	<0.001	24.8±7.1	42.0±6.8	<0.001	26.3±8.6	33.3±11.0	0.001	26.0±8.5	34.3±10.6	<0.001
Sokolow-Lyon voltage LVH	-	-	-	24 (9%)	36 (84%)	<0.001	42 (15%)	18 (47%)	<0.001	40 (15%)	20 (47%)	<0.001
Sokolow-Lyon product	2225±743	3888±759	<0.001	2260±694	4322±695	<0.001	2450±914	3194±1264	0.001	2376±862	3583±1121	<0.001
Sokolow-Lyon product LVH	7 (3%)	36 (60%)	<0.001	-	-	-	28 (10%)	15 (40%)	<0.001	23 (8%)	20 (47%)	<0.001
Cornell voltage (mm)	15.0±5.5	21.9±7.1	<0.001	15.4±5.6	21.6±8.5	<0.001	14.8±5.1	26.9±5.2	<0.001	14.6±4.8	26.8±5.3	<0.001
Cornell voltage LVH	20 (8%)	18 (30%)	<0.001	23 (8%)	15 (35%)	<0.001	-	-	-	13 (5%)	25 (58%)	<0.001
Cornell product	1660±553	2270±848	<0.001	1668±531	2458±960	<0.001	1618±480	2931±679	<0.001	1577±423	3036±510	<0.001
Cornell product LVH	23 (9%)	20 (33%)	<0.001	23 (8%)	20 (47%)	<0.001	18 (7%)	25 (66%)	<0.001	-	-	-

LV, left ventricle; IVS, interventricular septum; PW, posterior wall; LA, left atrium; RV, right ventricle; E/E', ratio of early mitral inflow velocity to peak mitral annulus velocity; LVH, left ventricular hypertrophy.

Data are presented as mean ± SD.

**Figure 1:**
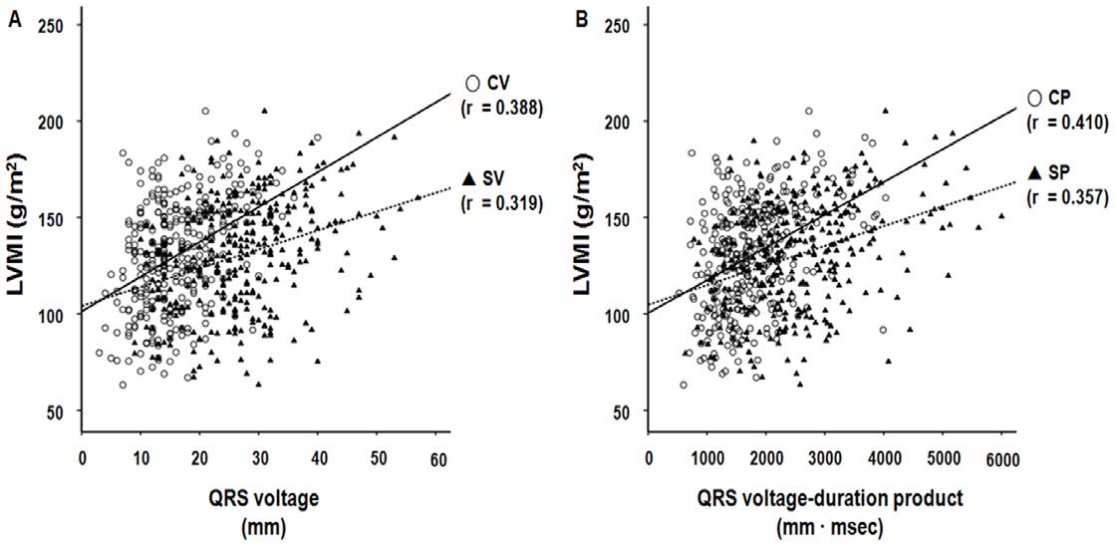
Correlation between electrocardiographic LVH and left ventricular mass index (LVMI). (A) Sokolow-Lyon voltage (SV), Cornell voltage (CV), (B) Sokolow-Lyon voltage-duration product (SP), and Cornell voltage-duration product (CP) correlated significantly with LVMI. Data are correlation coefficients (r).

**Figure 2:**
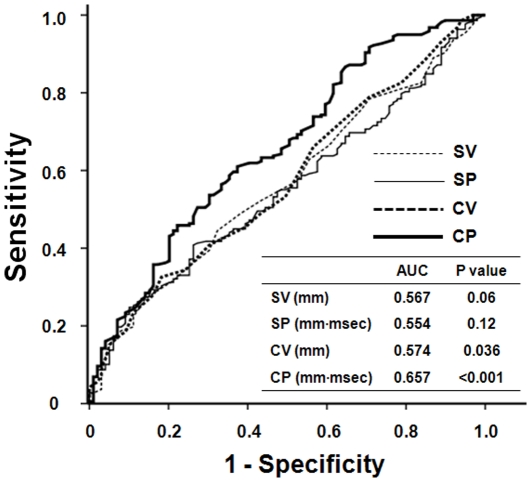
ROC curve analysis for echocardiographic LVH. The ROC curve was constructed by plotting the sensitivity (true positive rate) vs. 1-specificity (false positive rate) for each ECG-LVH criterion. At the highest predicted probability, sensitivities of Sokolow-Lyon voltage (SV), Sokolow-Lyon voltage-duration product (SP), Cornell voltage (CV), and Cornell voltage-duration product (CP) were 27.1%, 40.8%, 32.6%, and 45.9%, respectively.

Pearson's correlation analysis revealed that SP (r = 0.357, P<0.001) and CP (r = 0.410, P<0.001) criteria seemed to correlate more closely with LVMI compared to SV (r = 0.319, P<0.001) and CV (r = 0.388, P<0.001) criteria, respectively, but the differences did not reach statistical significance (SP vs. SV, Z statistic = 0.542, P = 0.59; CP vs. CV, Z statistic = 0.172, P = 0.86) (Figure 1). The positive predictive values of SP (86.0%) and CP (89.5%) for echocardiographic LVH were also higher relative to those of SV (81.7%) and CV (86.0%). Among the four ECG criteria, moreover, the CP criteria provided the highest predictive value for echocardiographic LVH in ROC curve analysis (AUC = 0.657, P<0.001) (Figure 2). Furthermore, the AUC of the CP criteria was significantly greater than those of the other three ECG-LVH criteria (CP vs. CV, Z statistic = 4.793, P<0.001; CP vs. SP, Z statistic = 2.707, P = 0.007; CP vs. SV, Z statistic = 2.146, P = 0.032).

### Clinical outcomes

**Figure 3:**
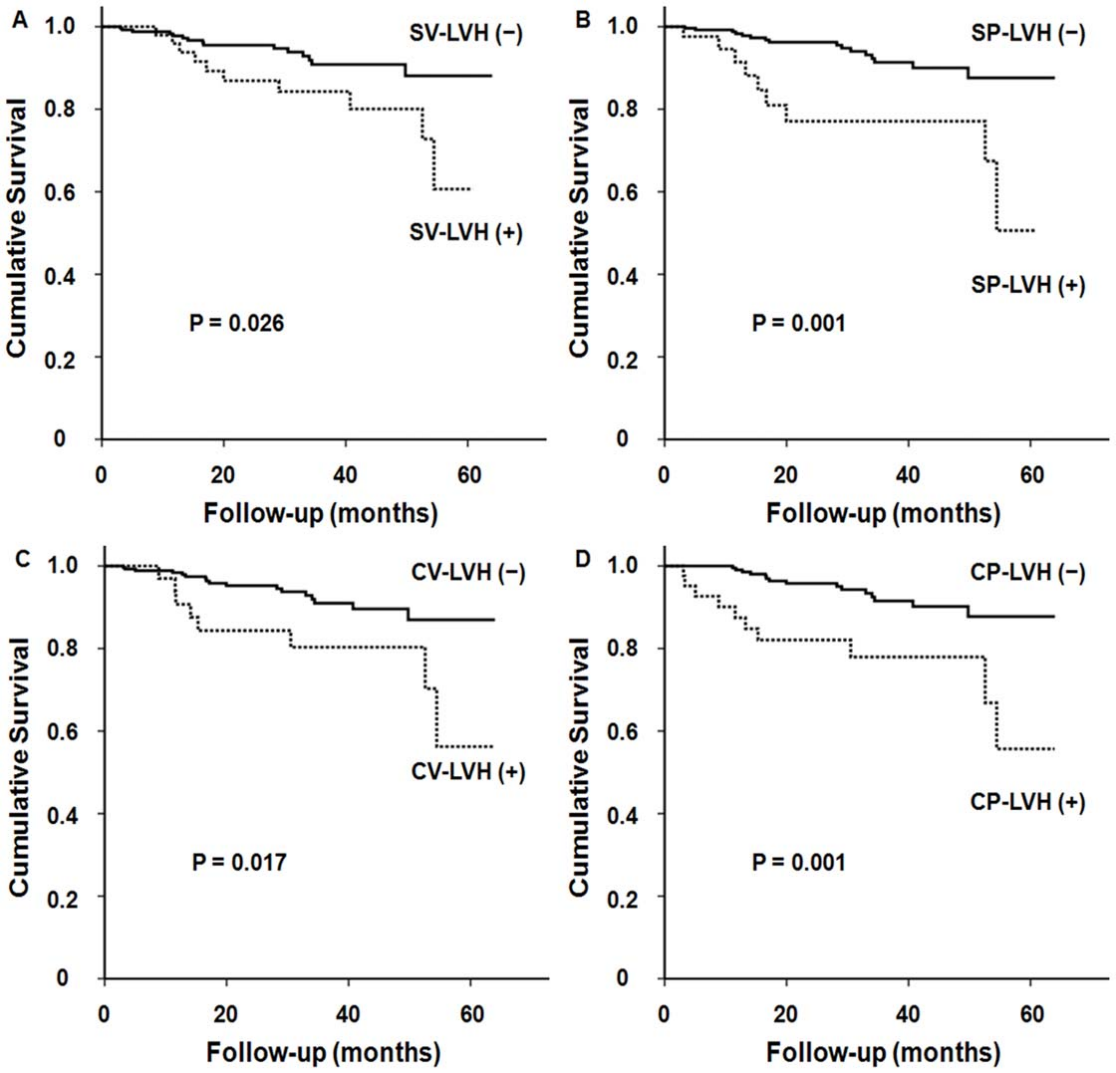
Kaplan-Meier curves for cardiovascular survival. Compared to patients without electrocardiographic LVH, the 5-year cardiovascular survival rates were significantly lower in patients with electrocardiographic LVH based on (A) Sokolow-Lyon voltage (SV), (B) Sokolow-Lyon voltage-duration product (SP), (C) Cornell voltage (CV), and (D) Cornell voltage-duration product criteria (CP).

During the mean follow-up duration of 27.4±17.2 months (3.0–64.0 months), 41 patients (12.9%) died over 725 patient-years of cumulative follow-up, yielding a crude mortality rate of 5.66/100 patient-years. Among them, 25 patients (7.9%) died from cardiovascular causes. Patients with echocardiographic LVH had a significantly lower cardiovascular mortality-free survival than those without echocardiographic LVH (72.2% vs. 98.0%, P = 0.003). In addition, the 5-year cardiovascular survival rates were significantly lower in patients with ECG-LVH by SV (60.7% vs. 88.1%, P = 0.026), SP (50.6% vs. 87.6%, P = 0.001), CV (56.2% vs. 87.0%, P = 0.017), and CP criteria (55.7% vs. 87.7%, P = 0.001) (Figure 3). Moreover, patients with ST-T wave abnormalities secondary to LVH, including a horizontal or downsloping ST segment and T wave inversion, showed significantly lower cardiovascular survival rates compared to those without these findings (39.5% vs. 87.3%, P = 0.005). However, there was no significant difference in cardiovascular mortality between patients with concentric and eccentric LVH and between patients with and without QTc interval prolongation. The overall mortality was also comparable between patients with and without echocardiographic LVH or ECG-LVH.

**Figure 4:**
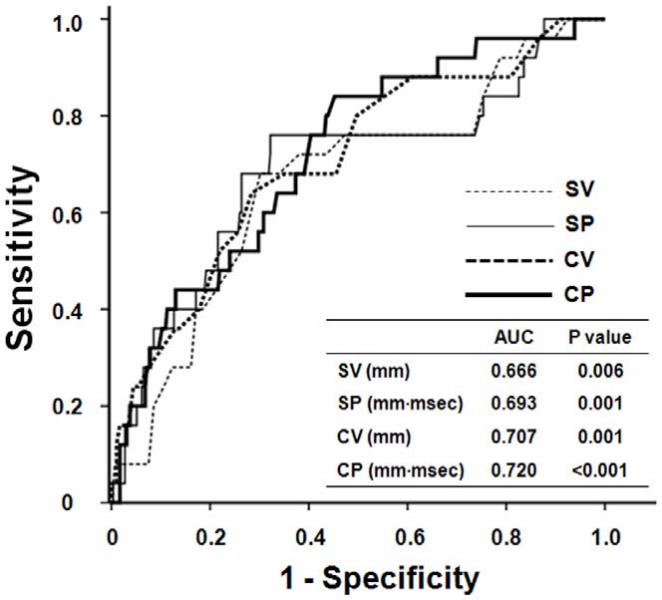
ROC curve analysis for cardiovascular mortality. The ROC curve was constructed by plotting the sensitivity (true positive rate) vs. 1-specificity (false positive rate) for each ECG-LVH criterion. At the highest predicted probability, sensitivities of Sokolow-Lyon voltage (SV), Sokolow-Lyon voltage-duration product (SP), Cornell voltage (CV), and Cornell voltage-duration product (CP) were 68.1%, 72.3%, 64.2%, and 76.0%, respectively.

Table 3 shows the hazard ratios (HRs) for cardiovascular mortality according to the presence of echocardiographic or electrocardiographic LVH. In an unadjusted Cox regression model, there was a significant increased risk for cardiovascular mortality in patients with echocardiographic LVH and ECG-LVH by each criterion. In multivariate analysis adjusted for age, diabetes, and coronary artery disease, which were revealed as significant independent predictors of cardiovascular mortality in univariate analysis, echocardiographic LVH and ECG-LVH based on QRS voltage-duration product were still significantly associated with cardiovascular mortality, but the significant association between ECG-LVH by purely voltage-based criteria and cardiovascular mortality in the unadjusted model disappeared (Model 1). Moreover, even when factors of specific interest, such as ejection fraction, ST-T wave changes, and QTc interval, were included in a multivariate model, echocardiographic LVH (HR: 11.71; 95% CI: 1.57–87.18; P = 0.016) and ECG-LVH by SP (HR: 3.43; 95% CI: 1.32–8.92; P = 0.011) and CP (HR: 3.07; 95% CI: 1.16–8.11; P = 0.024) criteria, but not SV and CV criteria, were significantly associated with cardiovascular mortality (Model 2). In ROC curve analysis, CP criteria provided the highest predictive value for cardiovascular mortality (AUC = 0.720, P<0.001), but there were no statistical differences in the AUC among the four ECG-LVH criteria (Figure 4).

**Table 3:** Cox regression models for cardiovascular mortality.

	unadjusted		Model 1		Model 2	
	HR (95% CI)	P-value	HR (95% CI)	P-value	HR (95% CI)	P-value
Echocardiographic LVH	10.88 (1.47–80.43)	0.019	9.11 (1.22–67.82)	0.031	11.71 (1.57–87.18)	0.016
Sokolow-Lyon voltage LVH	2.42 (1.08–5.42)	0.031	1.68 (0.71–3.97)	0.24	2.00 (0.86–4.68)	0.11
Sokolow-Lyon product LVH	3.75 (1.64–8.53)	0.002	2.80 (1.19–6.54)	0.018	3.43 (1.32–8.92)	0.011
Cornell voltage LVH	2.70 (1.16–6.31)	0.022	2.25 (0.95–5.30)	0.06	1.84 (0.68–4.97)	0.23
Cornell product LVH	3.69 (1.65–8.25)	0.001	2.64 (1.14–6.13)	0.024	3.07 (1.16–8.11)	0.024

Data are reported as hazard ratio (HR) and 95% confidence interval (CI).

Model 1: Adjusted for age, diabetes, and coronary artery disease.

Model 2: Adjusted for ejection fraction, ST-T wave abnormalities, and QTc interval.

## Discussion

In ESRD patients, LVH detected by ECG or echocardiography is the most common manifestation of cardiovascular disease and strongly predicts cardiovascular morbidity and mortality [2], [3], [7]–[9]. In this study, we demonstrate that SP and CP criteria correlated more closely with LVMI determined by echocardiography compared to SV and CV criteria, respectively, and that CP criteria provide the highest predictive value for identification of LVH. In addition, LVH based on QRS voltage-duration product is an independent predictor of cardiovascular mortality in incident HD patients, whereas LVH by QRS voltage-based criteria is not.

### Prevalence of LVH

LVH is prevalent in patients with CKD and its prevalence is known to increase as eGFR decreases [4], [5], [29], [30]. However, previous studies show wide variation in the prevalence of LVH in CKD and ESRD patients. A very recent study revealed that the prevalence of ECG-LVH by the Sokolow-Lyon criteria was 10% and by Cornell criteria was 14% in patients with CKD [24]. However, in a Spanish multicenter study on hypertensive patients, more than 20% of the subjects with CKD had ECG-LVH by Cornell criteria [22]. Meanwhile, Foley et al. demonstrated that LVH by echocardiography was present in 74% of ESRD patients at the start of dialysis [4], whereas Levin et al. found the overall prevalence of echocardiographic LVH to be 36% of ESRD patients [5]. In the 4D study, even though all patients were ESRD patients on hemodialysis and had type 2 diabetes, only 12.4% had EKG-LVH by Sokolow-Lyon criteria [31]. The results of the present study also revealed wide variation in the prevalence of LVH: 18.9% by SV, 13.6% by SP, 12.0% by CV, 13.6% by CP criteria, and 68.8% by echocardiography. We surmise that these discrepancies in the prevalence of LVH can be attributed to differences in patient age, gender, ethnicity, BMI, hemoglobin levels, and residual renal function and the proportion of patients with hypertension. Particularly, obesity has been shown to decrease the sensitivity of precordial lead ECG criteria, especially SV criteria, for the identification of LVH because QRS amplitudes are attenuated by interposed tissue, which increases the distance of exploring electrodes from LV [32]. In our study, the prevalence of ECG-LVH by SV criteria might in part be influenced by a relatively low BMI of the subjects, and the accuracy of SV criteria might be lessened because they were not gender-based. Whether LVH was assessed by echocardiography or ECG and which ECG criteria were used to define LVH may contribute to this wide variation in the prevalence of LVH.

### LVH and cardiovascular outcomes

Mounting evidence indicates that LVH is a powerful independent predictor of cardiovascular mortality in patients with CKD and ESRD [9]. Moreover, the change in LVH has been demonstrated as a strong prognostic factor in these patients. A previous prospective study on prevalent HD patients revealed that the rates of LVMI increase were significantly higher in patients with incident cardiovascular events than in those without such events, and that cardiovascular event-free survival in patients with changes in LVMI below the 25th percentile was significantly higher than in those with changes above the 75th percentile [8]. Similarly, in a cohort study of 153 incident ESRD patients receiving HD, a 10% reduction in LVM during a mean follow-up duration of 54 months resulted in a 22% decrease in all-cause mortality and a 28% decrease in cardiovascular mortality [7]. Furthermore, in that study, LVM regression was independently associated with improved patient survival even after adjustment for age, gender, diabetes, history of cardiovascular disease, and all nonspecific cardiovascular risk factors. While these two studies used echocardiography to assess LVMI or LVM as an indicator of LVH, similar results were observed in hypertensive patients with ECG-LVH [33], [34]. With a median interval of 23 days, persistent ECG-LVH at baseline and follow-up identified patients with greater LVM and a higher prevalence of echocardiographic LVH, suggesting that these patients may be at higher risk for subsequent morbidity and mortality [23]. We did not clarify the impact of the changes in LVM or LVMI by echocardiography, newly developed ECG-LVH, and the regression or persistence of ECG-LVH on patients' outcome. Nevertheless, consistent with most previous studies, the baseline echocardiographic LVH at the time of HD initiation was found to be significantly associated with worse cardiovascular survival in incident ESRD patients.

### ECG for the detection of LVH

ECG is a simple tool for the detection of LVH and is widely used in clinical setting [19]. According to the Kidney Disease Outcome Quality Initiative (K/DOQI) practice guidelines, recording of an ECG is recommended in every patient at the initiation of renal replacement therapy and yearly thereafter [35]. Due to the relatively low sensitivity of traditional ECG criteria for the detection of LVH, however, the clinical utility of ECG is limited [20]. On the other hand, prolongation of QRS duration is often observed in patients with LVH [36]. In addition, QRS duration was demonstrated to correlate with LVM [21]. Even though the mechanism for QRS prolongation in LVH has not been clearly determined, it may be related to a longer time required to activate myocardium, decreased conduction velocity in hypertrophic myocardium, and changes in activation sequence or in the relative conductivity of fibrotic intracellular and extracellular spaces [21]. In fact, the VIPE study, which evaluated the effect of candesartan-based treatment on LVH in hypertensive patients, demonstrated that candesartan treatment for 6 months not only reduced LVM, but also shortened the QRS duration [37]. Nevertheless, QRS duration alone has been proven to be poorly sensitive at clinically relevant levels of specificity [36]. For these reasons, there have been many attempts to increase the sensitivity of ECG for the identification of LVH by combining the voltage criteria with QRS duration, and these efforts have modestly improved the performance of ECG for detecting LVH in the general population and hypertensive patients [22]. However, most previous studies on CKD or ESRD patients used voltage criteria to determine ECG-LVH and did not validate ECG-LVH criteria for clinical outcomes [31]. Moreover, few studies have explored the relationship between electrocardiographic and echocardiographic LVH. In this study, we tried to elucidate the correlation between commonly used criteria of ECG-LVH and echocardiographic LVH, and found that SP and CP criteria correlated more closely with LVMI determined by echocardiography than SV and CV criteria, respectively and that CP criteria provided the highest predictive value for echocardiographic LVH. Furthermore, besides echocardiographic LVH, ECG-LVH only by SP and CP criteria was an independent risk factor for cardiovascular mortality in incident HD patients. These findings suggest that the considering QRS duration in addition to voltage may not only improve the identification of LVH but also serve as a more significant predictor of cardiovascular outcome in ESRD patients.

### Echocardiography for the detection of LVH

Echocardiography is a noninvasive procedure and provides an accurate assessment of ventricular size, geometry, and function. However, in ESRD patients, echocardiographic measurements, particularly of LVM, are highly dependent on the timing of echocardiography in relationship to dialysis sessions and to intravascular volume [38]. In addition, compared to MRI, echocardiography is known to significantly overestimate LVM in HD patients when LVH and dilation are present [17]. Moreover, the intra-observer and inter-observer variability of echocardiography are significantly higher than those of MRI. Nevertheless, MRI is not routinely performed because it is not widely available, is expensive, and cannot be used in patients with cardiac implantable devices. Consistent with most clinical and research studies, we used LVMI by echocardiography to determine LVH. Furthermore, since the ability to detect LVH in HD patients is improved by performing echocardiography on a nondialysis day, preferably between 12 and 18 hours after the last dialysis session, patients included in the present study underwent echocardiography on a nondialysis day, within 24 hours after the last dialysis, and near the time of discharge to minimize the volume effect on echocardiographic parameters.

### Limitations

Our study has several limitations. First, since the study subjects were all Korean ESRD patients on HD, the association between EKG-LVH criteria and clinical outcomes may not be generalizable to other populations. In addition, even though the number of patients was small (18 patients), patients with severe systolic dysfunction and severe valvular heart disease were excluded in this study because we inferred that these cardiac conditions *per se* could not only strongly affect the patient survival but also influence QRS voltage and duration. Nevertheless, we could not completely affirm that there was no selection bias. Second, the 5-year mortality rates in the present study were relatively lower compared to those of previous studies on Western ESRD patients [8], [30], [31], but they were comparable to those of Japanese patients on HD [29]. Moreover, a small number of events limited the power of the statistical analysis in identifying independent predictors of cardiovascular mortality. Therefore, only 3 factors could be evaluated at a time in the multivariate analysis to maintain the statistical power. Third, we analyzed only the ECG taken at the time of echocardiography. Thus, the possibility of intra-individual variability could not be completely excluded. However, the lower day-to-day variability that has been demonstrated for the measurement of QRS duration could enhance the general use of voltage-duration product criteria to lessen the influence of day-to-day variability [39]. Fourth, LVMI by echocardiography was regarded as the differentiating indicator of LVH in this study. As mentioned earlier, even though MRI is considered to be the “gold standard” technique for the assessment of LVM [17], we were unable to routinely perform MRI in all incident ESRD patients mainly due to its cost. Finally, follow-up ECG and echocardiography were not included for analysis in the present study. It would be worthwhile to investigate the impact of changes in ECG-LVH by different criteria on patient outcomes.

### Conclusions

This study shows that ECG-LVH based on QRS voltage-duration product predicts adverse cardiovascular outcomes better than ECG-LVH by QRS voltage-based criteria in incident HD patients. Our findings suggest that standard ECG itself may be of help in risk stratification and in providing therapeutic direction for the management of these patients.
